# Life-quality of orthognathic surgery patients: The search for an integral diagnosis

**DOI:** 10.1590/2176-9451.19.1.123-137.sar

**Published:** 2014

**Authors:** José Augusto Mendes Miguel, Nathália Barbosa Palomares, Daniela Feu

**Affiliations:** 1 Adjunct professor, State University of Rio de Janeiro (UERJ).; 2 Masters student of Dentistry, UERJ.; 3 Adjunct professor of Orthodontics, Vila Velha University (UVV).

**Keywords:** Quality of life, Orthodontics, Orthognathic surgery

## Abstract

The decision on whether starting an orthosurgical treatment depends on the negative esthetic, functional and social impact the dentofacial deformity has on the quality of life of each patient. The objective of this article is to demonstrate the importance of assessing the quality of life of these individuals by means of applying specific questionnaires before treatment onset in order to increase the success rate of orthosurgical treatment. These questionnaires assess not only the esthetic factor, but also the functional conditions that may be affected as well as the psychological issues related to self-esteem and sociability, all of which must be assessed in order to enable the development of an individual treatment plan that meets patient's expectations. Thus, a more predictable level of satisfaction can be achieved at treatment completion, not only from a normative standpoint stated by professionals, but also from a subjective standpoint stated by patients. Although not enough comparable data is available in the literature for us to assess the extent of improvements produced by orthosurgical treatment, a few recent reports conducted by different universities around the world reveal a good response from the majority of patients after surgery, demonstrating great satisfaction with regard to esthetic, functional and psychosocial factors. Therefore, it is reasonable to conclude that the current objective of orthodontic treatment associated with orthognathic surgery consists not only in treating the esthetic functional components of dentofacial deformities, but also in considering patients' psychological factor.

## Abstract (pt)

A decisão de iniciar um tratamento ortodôntico-cirúrgico depende do impacto negativo estético, funcional ou social gerado pela deformidade dentofacial na qualidade de vida de cada paciente. O objetivo deste artigo é demonstrar a importância da avaliação da qualidade de vida desses indivíduos para elevar a taxa de sucesso no tratamento ortodôntico-cirúrgico, por meio da aplicação de questionários específicos antes do início do tratamento. Esses instrumentos avaliam, além do fator estético, as condições funcionais que podem estar afetadas e os problemas psicológicos relacionados à autoestima e à sociabilidade, que devem ser identificados para possibilitar a elaboração de um plano de tratamento individualizado para as expectativas subjetivas do paciente. Assim, torna-se mais previsível obter um alto grau de satisfação ao final do tratamento ortodôntico-cirúrgico, tanto do ponto de vista normativo avaliado pelos profissionais quanto do ponto de vista subjetivo dos pacientes. Apesar da deficiência de dados comparáveis suficientes na literatura disponível para avaliar a extensão da melhora após o tratamento ortodôntico-cirúrgico, há relatos recentes de universidades em diversos países que demonstram uma boa resposta da maioria dos pacientes após a cirurgia, com alto grau de satisfação dos pontos de vista estético, funcional e psicossocial. Conclui-se que o objetivo atual do tratamento ortodôntico associado à cirurgia ortognática consiste em não apenas tratar os componentes estético e funcional da deformidade dentofacial, mas, também, o de considerar o componente psicológico do paciente.

## INTRODUCTION

Facial esthetics strongly influences personal and professional relations, especially in school and professional environments, from childhood to adulthood.^1,2,3^ Patients with severe malocclusions are dissatisfied with their physical appearance, particularly with their face.^4^ In cases of dentofacial deformities, in which patients wish to significantly change their face and solve their functional problems, orthosurgical treatment is the most suitable option.

Although the vast majority of published articles only highlight the surgical techniques available, assessing the effects of dentofacial deformities and orthosurgical treatment on the psyche of each patient is essential. Additionally, it is highly necessary that psychological and functional issues, social interaction trouble, low self-esteem and other negative impact that hinder patient's quality of life be identified.^1,5,6^

The concept of "quality of life" was defined in 1993 by the World Health Organization as the perception of people with regard to their situation in life, within the cultural context and values with which they live, in relation to their objectives, expectations, patterns and concerns.^7^ Quality of life is essentially a subjective concept that cannot be judged by others.

According to the current paradigm of evidence-based Dentistry, all treatment procedures must be based on the systematic assessment of clinically relevant scientific evidence available which include patients' current condition, medical/dental history, treatment needs and preferences. Although the demand for orthosurgical treatment is strongly related with patients' chief complaint about their appearance, as well as with psychological and social interaction issues, assessments on the need for treatment give little emphasis on patients' perception and on how much treatment can improve their oral health-related quality of life.^8^

The ideal would be to implement objective clinical indexes as well as subjective indexes assessing the impact of dentofacial deformities on the daily routine of affected individuals. The results would reflect patients' demand and guide the priorities of public healthcare systems. Private practice allows orthodontists and dental surgeons to have a better understanding of patients' chief complaints and expectations with regard to treatment outcomes, allowing professionals to develop more personal treatment plans, with higher levels of foreseeability regarding patients' satisfaction at treatment completion.^9^

### Motivations of patients who seek orthosurgical treatment

Over the years, studies have demonstrated that most patients with dentofacial deformities seek treatment in order to have their facial and dental esthetics improved.^10^ Additionally, some studies report that the main motivation comprises improvements in masticatory function rather than changes in appearance.^11^ Patients also seek treatment with the expectation of gaining psychosocial benefits, including improvements in interpersonal relationships and psychological well-being, by improving their self-esteem.^12^

Some patients may have unreal expectations with specific objectives such as professional growth or romantic relationships. These cases are often related with previous frustrating experiences which the individual relates to the presence of his dentofacial deformities. These patients overestimate the influence this treatment has over their lives,which must be identified during the first interview conducted by both orthodontist and dental surgeon in order to prevent a possible misunderstanding between professionals and patients.

In spite of that, the most common situation identified in epidemiologic research as well as in orthodontic clinics is that patients expect to have improvements in their psychological well-being and their interpersonal relationships without directing their expectations towards unreal situations.

Available scientific literature investigating the theme agrees that patients believe that their lives will improve after orthodontic treatment.^10,12^

Therefore, patients' subjective expectations, which considerably differ from those of orthodontists as well as from oral and maxillofacial surgeons, must be investigated before any intervention is carried out.^1,5,8,12-18^

### How to evaluate oral health-related quality of life?

Quality of life instruments can be addressed by direct or telephone interviews, self-filling questionnaires or, should the individual not be able to answer the questions himself, questionnaires filled in by other people.^19^ The most widely used method is the questionnaire filled in by the patient himself, given that this method reduces the chances of interference. Each questionnaire focuses on a different aspect of quality of life evaluation: General health, oral health or specific health according to the condition under study (for instance, orthosurgical treatment).

The Medical Outcomes Study 36-Item Short-Form Health Survey (SF-36) is a general quality of life instrument that assesses the impact of a problem, treatment or intervention over patients' general health perception. It comprises psychiatric questions of more widespread and less specific perception, and is mainly used by Medical Sciences to compare different populations, although its use is limited in Dentistry. Its 36 items assess 8 domains divided into two groups: Physical (functional capacity, physical aspects, pain and general health status) and mental (mental health, vitality, as well as psychosocial and emotional aspects). Its scoring varies from 0 (no negative impact on quality of life) to 100 (the worst quality of life possible).^19^

**Figure 1:**
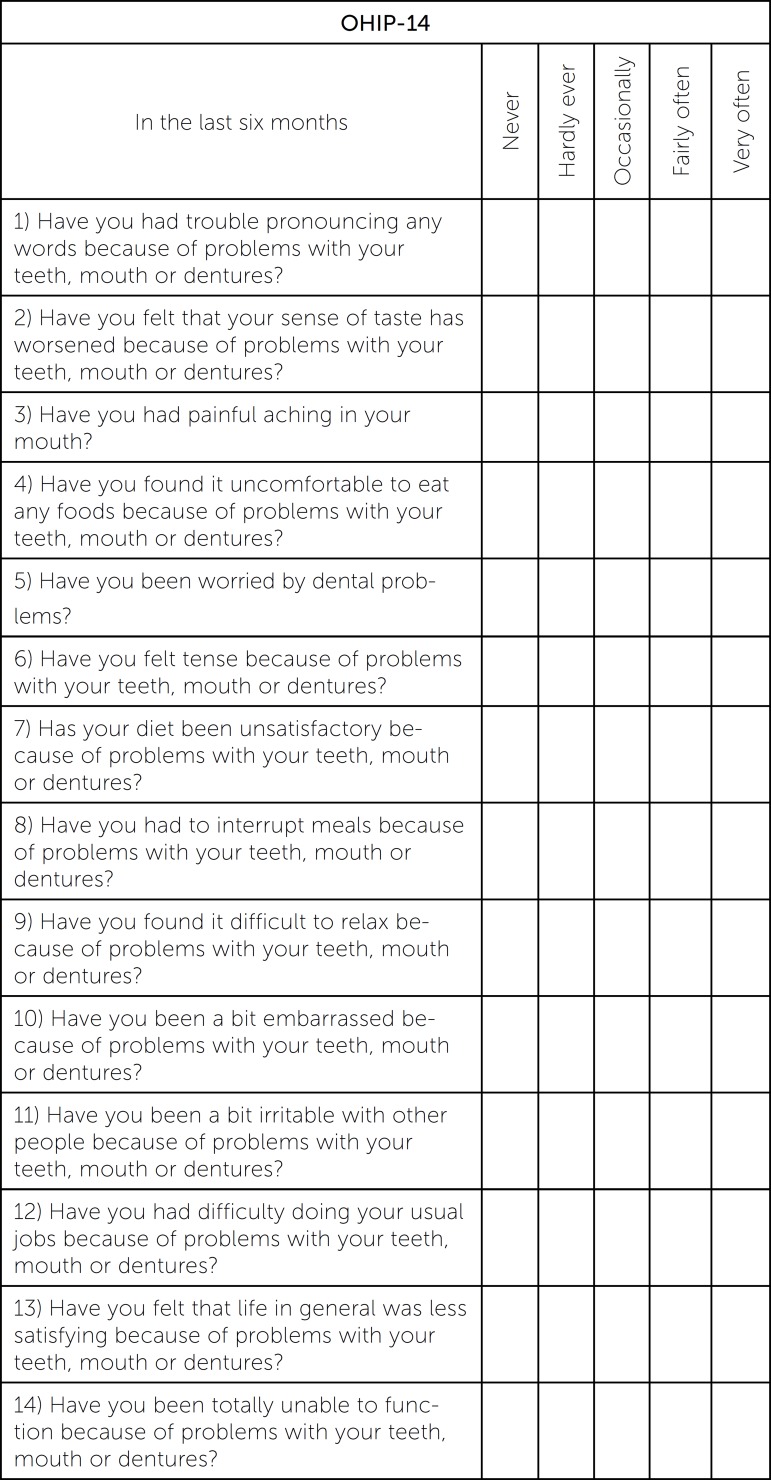
Oral health impact profile questionnaire: Short version (OHIP-14).

Among the questionnaires used to assess the impacts on oral health-related quality of life, the most widely used is the Oral Health Impact Profile (OHIP) developed in Australia by Slade et al,^20^ and which assess the individual's perception regarding discomfort and dysfunction caused by oral conditions. Its 49 items are divided into seven dimensions: Functional limitation, physical pain, psychological discomfort, physical incapacity, psychological incapacity, social incapacity and difficulty doing usual jobs. Its short version, known as OHIP-14, was published in 1997^21^ and comprises fourteen questions that assess the same seven dimensions (Fig 1). The interviewee must score points to each question according to the frequency with which he is affected: 0 = never; 1 = hardly ever; 2 = occasionally; 3 = fairly often and 4 = very often. The sum of points for the 14 questions gives the final OHIP-14 score which may vary between 0 and 56, in which 0 means absence of negative impact and 56 means the worst negative impact on oral health-related quality of life. The Brazilian version of the OHIP-14 proved to have psychometric properties that are similar to the original questionnaire.^22^

**Figure 2:**
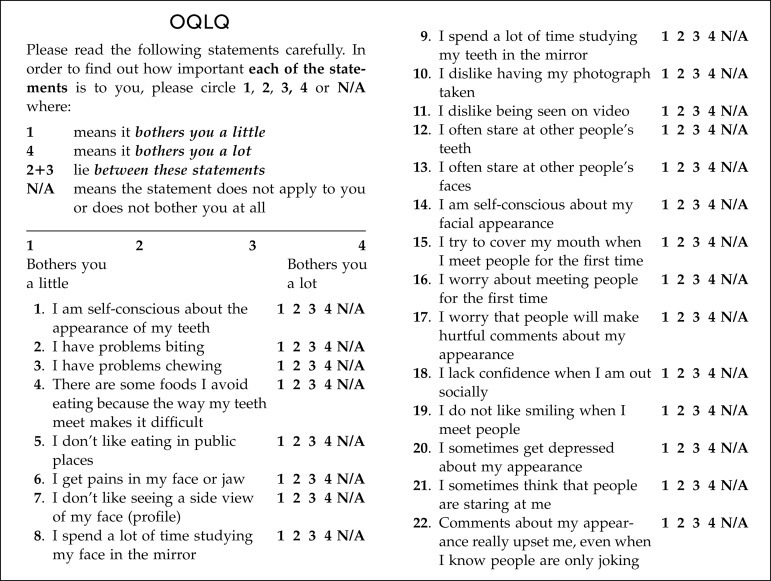
Quality of life questionnaire for orthodontic-surgical patients (OQLQ).

The Orthognathic Quality of Life Questionnaire (OQLQ) was developed and validated by Cunningham et al^23,24^ whose objective was to assess the impact of dentofacial deformities and the benefits of orthosurgical treatment on patients' quality of life (Fig 2). This questionnaire has been widely used in researches^14,16,18^ and comprises 22 questions divided into four domains: Facial esthetics, oral function, awareness of facial esthetics and social aspects. Patients can choose the option "it does not bother me" (0 points) or, should they be affected by any of the issues, they use a 4-point scale of which answers vary from "it bothers me a little" (1 point) to "it bothers me a lot" (4 points). OQLQ total score can vary from 0 to 88. A lower score suggests improvements in quality of life, whereas a higher score suggests that the quality of life has become worse. The OQLQ has already been translated into Brazilian Portuguese^25,26^ with its psychometric properties being kept. Thus, the B-OQLQ is an instrument that must be used when assessing Brazilian orthodontic-surgical patients.

### How is the quality of life of patients with dentofacial deformities?

Combined orthodontic and orthognathic surgery therapy is the most appropriate treatment option for patients with facial deformities, given that it allows facial harmony to be established by surgical repositioning of jaw bones. Another treatment option includes orthodontic camouflage, however, this therapy only acts in dental positioning and inclination, improving intercuspation without significant changes in facial esthetics.

Improvements in facial esthetics have been considered the main motivating factor for patients seeking orthosurgical treatment.^27^ Such desire is explained by the role esthetics plays in interpersonal and professional relationships,^19^ which suffer social and psychological implications when facial deformities are present.^18^ In other words, patients with dentofacial deformities are subject to prejudgment that may hinder their social relations and influence their self-body image.^12^

Self-body image comprises two elements: The first is the individual's self-image seen in a mirror or in a photograph; while the second, and most important, is how the individual feels towards his features. Patients with a positive self-body image have higher chances of presenting more realistic expectations towards the outcomes of corrective procedures to which they will undergo in comparison to patients with a negative self-image.^17^ It is essential that clinicians clearly understand the expectations of patients who seek orthosurgical treatment, given that such therapy causes significant changes in individuals' body image. Should the dental surgeon and the orthodontist in charge be unaware of patients' expectations, or should any desires related to the therapy not be accomplished by the end of treatment, the outcomes may end up being very frustrating, even if the objectives initially set by the professionals, by means of normative indexes, are achieved.^12,17^

That is how the application of specific questionnaires aimed at assessing orthodontic-surgical patients' quality of life in clinical practice can contribute to diagnosis and treatment planning. These instruments assess not only esthetic aspects, but also functional conditions that may have been affected (for instance, mastication, speech and breathing) as well as psychological issues related to self-esteem and sociability.^17^

### Impact of orthosurgical treatment on quality of life

Recent reports focusing on patients' satisfaction demonstrate good response after surgery, which has been confirmed by many authors.^1-3,5,6,12,15,16,18^ Nevertheless, lack of comparable data to assess the extent of improvements produced by orthosurgical treatment has been one of the main issues with regard to the available literature. This is mainly due to lack of consensus between the different indexes used to assess such changes.^27^ Table 1 lists the clinical studies^5,8,15,16,24,28-37^ that assessed the effects of orthosurgical treatment on the quality of life of patients with dentofacial deformities (Table 1).

**Table 1:** Clinical studies assessing the effects of orthosurgical treatment on the quality of life of patients with dentofacial deformities.

Authors	Type of study	Period of assessment	Instruments used	n	Objectives	Main conclusions
Hatch et al^28^	Multicenter randomized clinical trial	Immediate pre and post-operative phase	Sickness Impact Profile, Oral Health Status Questionnaire, Symptom Checklist 90 Revised	117	Assess the effects of orthosurgical treatment on QoL and OHRQoL	QoL and OHRQoL of patients subjected to orthosurgical treatment significantly improved
Hatch et al^29^	Multicenter randomized clinical trial	Immediate pre and post-operative phase, six months and two years after surgery	Sickness Impact Profile, Oral Health Status Questionnaire, Symptom Checklist 90 Revised	117	Assess the effects of orthosurgical treatment on QoL and OHRQoL	QoL and OHRQoL significantly improved after orthognathic surgery and the results remained after a two-year follow-up
Cunningham et al^24^	Longitudinal prospective study without control	Before treatment, before surgery and 8 weeks after treatment completion	OQLQ and SF-36	55	OQLQ validation	OQLQ is a valid instrument that demonstrates improvements in OHRQoL of patients subjected to orthosurgical treatment
Motegi et al^30^	Multicenter randomized clinical trial	Immediate pre and post-operative phase, two and five years after surgery	Sickness Impact Profile, Oral Health Status Questionnaire, Symptom Checklist 90 Revised	93	Assess the maintenance of effects of orthosurgical treatment on QoL and OHRQoL	QoL and OHRQoL of patients subjected to orthosurgical treatment remained significantly improved two and five years after surgery (follow-up period)
Nicodemo et al^31^	Longitudinal prospective study without control	Before and after surgery	SF-36	29	Assess the effects of orthosurgical treatment on QoL	Treatment significantly improved patient's QoL in physical and social aspects
Lee et al^5^	Longitudinal prospective study without control	Before and after surgery	SF-36, OHIP-14 and OQLQ	36	Understand the changes in OHRQoL of orthosurgical patients	Treatment significantly improved patients' OHRQoL in spite of temporary post-surgical worsening
Al-Ahmad et al^32^	Control case	Before treatment, before and after surgery	SF-36 and OQLQ	143	Assess the impact of orthosurgical treatment on three groups of patients in different phases	The study suggests that orthosurgical treatment positively affects QoL and OHRQoL
Choi et al^15^	Longitudinal prospective study without control	Before treatment, before surgery, 6 weeks and 6 months after surgery and treatment	OHIP-14 and OQLQ	36	Understand the changes in OHRQoL and QoL of orthosurgical patients	Treatment significantly improved patients' OHRQoL in spite of temporary post-surgical worsening
Esperão et al^8^	Cross-sectional study without control	Before treatment, before and after surgery	OHIP-14	117	Assess the impact of orthosurgical treatment on three groups of patients in different phases	The study suggests that orthosurgical treatment positively affects OHRQoL
Khadka et al^16^	Longitudinal prospective study without control	Before and after surgery	SF-36 and OQLQ	152	Understand the changes in OHRQoL and QoL of orthosurgical patients	Improvements in OHRQoL and QoL are significantly higher for patients with functional and esthetic complaints
Murphy et al^33^	Longitudinal prospective study without control	Before and after surgery	OQLQ, Visual Analog Scale and Global Transition Scale	52	Understand the changes in OHRQoL of orthosurgical patients	Patients presented positive improvements in facial appearance, oral function and self-esteem
Ballon et al^34^	Retrospective study without control	Before surgery, 8 weeks and one year after surgery	OHIP-14, OQLQ, Zung Depression Scale and Rosemberg Self-esteem Questionnaire	45	Understand the changes in OHRQoL, self-esteem and symptoms of depression in patients subjected to orthosurgical treatment	Orthosurgical treatment did not significantly influence any of the assessed items
Rustemeyer and Gregersen^35^	Longitudinal prospective study without control	Before treatment and 12 months after surgery	OHIP-14	50	Understand the changes in OHRQoL of orthosurgical patients	Patients presented functional and psychological benefits after treatment
Rustemeyer et al^36^	Longitudinal prospective study without control	Before and after surgery	OHIP-14	30	Assess the changes in OHRQoL before and after surgery, associating them with cephalometric changes in hard tissues	Reduction in lip-chin angle and nasion-pogonion distance as well as increase in facial convexity led to significant reduction/improvements in OHIP-14 scores
Kavin et al^37^	Longitudinal prospective study without control	Before surgery, 8 and 24 weeks after surgery	OHIP-14 and OQLQ	14	Understand the changes in OHRQoL of orthosurgical patients	Treatment significantly improved patients' OHRQoL after 24 weeks in spite of temporary post-surgical worsening after 8 weeks
Soh and Narayanan^27^	Systematic literature review	_	Patients' QoL and psychosocial analysis instruments	19 papers	Understand the scientific evidence available for orthosurgical treatment and QoL	Patients' QoL and psychosocial aspects improved with orthosurgical treatment. A study by Motegi et al43 is the best evidence available

By applying the OHIP-14 questionnaire, Esperão et al^8^ observed that non-treated individuals seeking orthosurgical treatment suffered 6.5 times more negative impact on their quality of life than patients who had received orthosurgical treatment. In spite of presenting progressive worsening of occlusal aspects, patients who were undergoing orthodontic surgical preparation already presented improvements in quality of life, with three times more negative impact than operated patients. It is worth noting that women were the most affected in the pre-treatment phase.

These results were similar to those obtained by Rusanen et al^4^ who also detected by means of the OHIP-14 questionnaire that patients with facial deformities had more impact on their oral health-related quality of life than the general population, out of which female patients cared more about the opinion of other people.

Assessment of 93 Class II orthosurgical patients revealed significant improvements in quality of life during orthodontic preparation and post-surgical follow-up phases. Comparison of data obtained 2 years before and 5 years after surgery revealed no significant changes, thus demonstrating stability of patients' quality of life. The psychosocial dimension was more positively affected than physical and functional aspects.^30^

A prospective analysis of changes in the quality of life (assessed by OQLQ and OHIP-14) of 36 Class III malocclusion patients subjected to orthosurgical treatment^7^ revealed progressive reduction in OQLQ scoring six weeks after surgery, six months after surgery and at treatment completion.

However, OHIP-14 scoring revealed an important reduction only six weeks and six months after surgery. General quality of life, assessed by means of SF-36, also revealed significant improvements in mental health six months after surgery as well as in physical aspects six weeks after surgery. Thus, orthosurgical treatment was considered effective, producing significant psychosocial and functional improvements for patients.^15^

Lee et al^5^ also observed significant decrease in patients' general quality of life during the first six weeks after orthognathic surgery. Nevertheless, such decrease was transitory, and six months after surgery patients presented significant improvements.

A prospective analysis of 65 English patients who underwent orthosurgical treatment demonstrated significant gain in oral health-related quality of life, even during orthodontic preparation for surgery, despite the fact that the occlusal aspects progressively worsened. In the post-operative phase, oral health-related quality of life considerably improved. The most affected dimensions were: Social aspects, dentofacial esthetics and masticatory function.^24^

In the United States, Flanary et al^18^ observed significant gain in patients' self-concept and self-image after orthosurgical treatment, with reduction in the incidence of personality disorders, psychosis and neurosis. Such gain remained even two years after surgery.

In Brazil, Costa et al^3^ corroborated these results by observing significant improvements in self-image and self-esteem of 15 patients who underwent orthognathic surgery. The majority of patients assessed sought treatment after being referred by other dentists or orthodontists. Family psychological support was of paramount importance for patients' recovery, and after surgery, patients observed major changes in their facial features as well as in their self-esteem. Most esthetic and functional problems reported by patients were corrected after surgery.

Orofacial pain reports are usually associated with skeletal disharmony. Patients subjected to orthosurgical treatment reported improvements in masticatory function, speech and facial esthetics as well as reduction in articular and myofacial pain.^3^

Alves e Silva et al^6^ assessed the level of satisfaction of surgery performed in 15 orthosurgical Brazilian patients aged between 17 and 35 years old. Most patients (93%) claimed that surgery met their expectations, 33% had complaints with regard to the post-operative phase, especially in the first 24 hours after surgery (67%), whereas 60% complained about their eating habits in the first week after surgery (47%) and during maxillomandibular block with rubber bands (33%). As for social relations, 60% mentioned that they did not change, while 20% reported a slight increase and 20% reported a great increase.

Murphy et al^33^ used the Global Transition Scale (GTS) to assess whether patients' conditions improved or worsened after surgery. Patients who underwent surgery reported to have improvements in the following aspects: Facial appearance (93%), masticatory function (64%), oral health welfare (60%) and speech (32%).

According to evidence available, it is clear that self-image continues to be positively affected during a long period of time after orthosurgical treatment.^3,12^ Nevertheless, improvements in self-image tend to decline when orthodontic finishing lasts for more than nine months. After this period, patients have already recovered from surgery and present functional as well as nutritional improvements. However, feeling that the treatment is incomplete causes some discomfort, which may influence the benefits obtained and negatively affect patients' quality of life. Despite being temporary, negative feelings originating from presurgical orthodontic preparation are significant issues with which patients must deal with. In addition, they are considered the worst disadvantage of this type of treatment, given that they cause patients' esthetic and functional conditions to worsen.^8,12,13^

In conventional orthosurgical treatment, the presurgical orthodontic preparation phase is considered the worst aspect of treatment. It requires dental tipping decompensation and adaptation, in addition to significantly worsening patients' dentofacial aspects, thus producing negative esthetic and social impact and, as a consequence, worsening the social conditions already observed before treatment onset, which may lead to an intense feelings of disadvantage.^8^

This preparation phase lasts for about 17 months, but may be carried out for a period not greater than two years.^38^ It results in an ideal decompensation of dental tipping, space consolidation and coordination between the arches, thus allowing the best skeletal correction as well as the greatest possible stability.^39^ Once an appropriate, precise and stable occlusion has been achieved in order not only to allow the orthognathic surgery to be carried out, but also perfectly align the arches, only minor adjustments are left for the postsurgical phase.^38^

According to the literature, the postsurgical phase of conventional treatment lasts between seven and twelve weeks, and no changes regarding patients' age, sex and type of malocclusion were found to significantly change this duration.^38^ For Kiyak,^12^ the postsurgical treatment phase should not last for a period longer than nine months, since it could impair patients' psychological improvements.

According to Hernández-Alfaro et al,^40^ the presurgical preparation phase may be even more harmful for patients with Class III malocclusion, given that mandibular prognathism results in dental decompensation that strongly emphasizes skeletal disharmony in these patients. In spite of that, patients with Class III malocclusion tend to prevail among Brazilian patients with skeletal malocclusion who seek orthodontic treatment.^14^

Some authors observed improvements during the preparation phase, however, their data have not been completely explained. For instance, by conducting a prospective analysis, Cunningham et al^24^ concluded that orthosurgical treatment resulted in significant gains in OHRQoL of patients with dentofacial deformities. Patients' presurgical analysis revealed that a significant gain was obtained during presurgical orthodontic preparation in comparison with the results of the first examination, although the occlusal aspects progressively worsened. The second analysis was carried out six to eight weeks after orthodontic treatment finishing and, consequently, after orthognathic surgery was performed and the appliance was removed. It revealed significant improvements in oral health-related quality of life, which were greater than those found in previous analyses. The aspects that contributed the most for such important improvements in OHRQoL were: Social aspects, esthetics and masticatory function.

Despite being temporary, negative feelings originating from presurgical orthodontic preparation are significant issues with which patients must deal with. In addition, they are considered the worst disadvantage of this type of treatment, given that they cause patients' esthetic, functional and social conditions to become considerably worse than their initial malocclusion, the reason why they sought treatment.^8,13,42^

Nevertheless, the impacts must cease to exist if the benefits obtained with surgery can be achieved within a shorter period of time by the "Anticipated Benefit" protocol.^13,41^ This protocol aims at foreseeing the magnitude of the skeletal changes that are necessary for treatment to be carried out, with orthognathic surgery being performed right after the orthodontic appliance has been placed. Orthognathic treatment (arch alignment and leveling) can only be performed after orthognathic surgery has been carried out. Theoretically, the tension and anxiety of going through orthodontic surgery preparation would cease to exist and the patient would experience the benefits of treatment more quickly, however, specific studies are warranted to confirm such hypothesis.

Feu^14^ compared the effects of two orthosurgical treatment protocols on the quality of life of 16 patients with Class III skeletal malocclusion during two years. The protocols were equally divided into two groups: Conventional treatment and anticipated benefit protocol. The main finding of this research was that the quality of life and esthetic self-perception of patients comprising the groups treated by the anticipated benefit protocol were significantly superior to those comprising the conventional treatment group during all assessment periods. Data were first assessed one month after treatment had been performed, during a two-year follow-up. These data revealed that, to date, treatment performed with the anticipated benefit protocol has a more positive psychosocial impact than conventional orthosurgical treatment. After two years, patients comprising the anticipated benefit protocol group were either undergoing the final stages of orthodontic finishing or had treatment already completed, and were then in retention. Conversely, none of the patients comprising the conventional group had been subjected to surgery and their quality of life had considerably become worse due to extended preparation time and worse occlusal conditions. It is still unknown whether or not, after surgery, patients comprising the conventional treatment group will have quality of life scores and esthetic self-perception similar or better than those of patients comprising the anticipated benefit protocol group, given that their occlusal condition will be better after surgery due to presurgical orthosurgical treatment preparation.

According to evidence available, patients with dentofacial deformities present significant improvements in self-image after orthosurgical treatment, which continues to be positively affected during a long period of time after orthosurgical treatment.^42^ Nevertheless, improvements in self-image tend to significantly decline when orthodontic finishing lasts for more than nine months, in which case treatment seems to be incomplete. As reported by Kiyak et al,^12^ in these cases, patients are no longer satisfied with treatment outcomes and present a reduction in their self-esteem and self-image, which theoretically is a result of losing psychological welfare. After this period of nine months, patients have already recovered from surgery and present functional as well as nutritional improvements, however, feeling that the treatment is incomplete causes discomfort, which may influence the benefits obtained.^12,43^ Additional studies are warranted to confirm whether or not these effects affect patients' OHRQoL.

Likewise, after six months, patients subjected to conventional orthosurgical treatment had mandibular movements as well as condylar displacement fully recovered.^44^ These are important indicators of masticatory efficiency and adjustment of the temporomandibular joint which, in turn, may be related to the functional dimensions of the quality of life questionnaires, although no scientific evidence has yet proved such a fact.^45^ Additional studies are warranted to further investigate the masticatory efficiency of patients treated with the anticipated benefit protocol. Therefore, assessing the functional dimensions of quality of life may provide important information on the adaptation of these patients during the postsurgical phase.

Longitudinal studies conducted with patients subjected to orthodontic treatment, regardless of the period of assessment, revealed that worse occlusal relationships were significantly related to a worse perception in oral health-related quality of life.^46,47^ In case of patients treated with the anticipated benefit protocol, even after skeletal relationships and unpleasant esthetics had been corrected, patients' occlusion remained unbalanced and their masticatory function stability became worse.^13^ Therefore, additional studies are necessary to further investigate patients' perception in this new treatment condition, not comparable to any other type of treatment that has been previously investigated.

## CASE REPORTS

Due to being an elective procedure, the decision of undergoing an orthosurgical treatment not only depends on patients' opinion, but also on their family's and on negative impact of the dentofacial deformity, whether esthetic, functional or social. For this reason, patients' expectations play an important role in predicting treatment final outcomes, given that their satisfaction is theoretically related to reduction or elimination of the factors that led them seek treatment. Thus, how can orthodontists or oral and maxillofacial surgeons be successful in performing an orthosurgical treatment that results in psychosocial gains for patients without knowing the impact caused by their dentofacial deformity?

Three cases of orthodontic-surgical patients treated at the Orthodontic Clinic of the State University of Rio de Janeiro, including normative and quality of life data, are reported herein. They demonstrate how joined analysis allows a more individualized treatment conduct to be carried out.

### Case report 1

**Figure 3:**
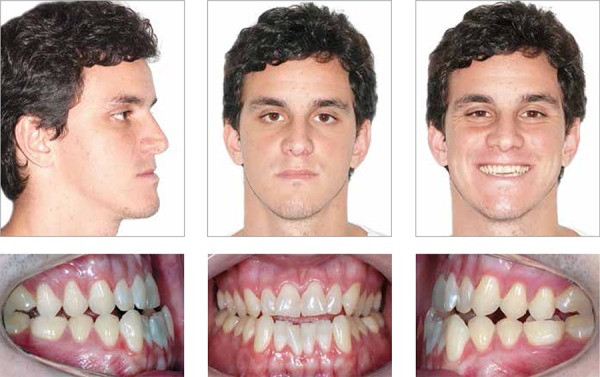
Initial facial and intraoral photographs.

Caucasian, male, 22-year old patient sought treatment complaining about his dentofacial esthetics. As shown in his initial photographs (Fig 3), he presented Class III malocclusion, maxillary deficiency and mandibular excess.

His oral health-related quality of life was assessed by means of the OHIP-14 which scored 31 points (an index that varies from 0 to 56 points), and the B-OQLQ which scored 73 points (from 0 to 88 points), thus suggesting a considerably negative impact. For this reason, orthosurgical treatment was planned with the anticipated benefit protocol^13^ in order to quickly improve patient's facial esthetics.^15,18^

An orthodontic appliance was placed and surgery associating maxillary advancement and mandibular setback was performed immediately after that.

**Figure 4:**
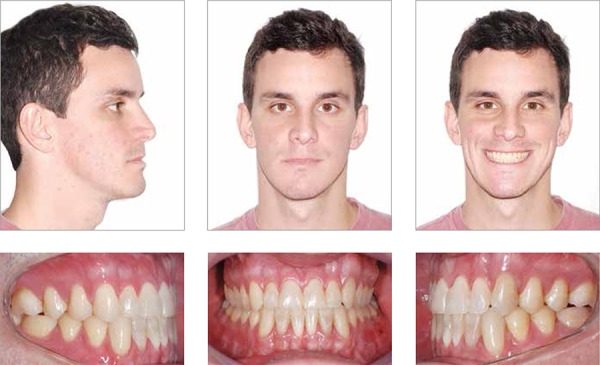
Final facial and intraoral photographs.

After two years and seven months of treatment, the orthodontic appliance was removed. Satisfied with the final outcomes and the dental alignment obtained (Fig 4), the patient scored 4 points in the OQLQ and 4 points in the OHIP-14, which means he presented extremely significant improvements in oral health-related quality of life. In this case, the correct diagnosis of objective clinical data and patient's subjective perception led to successful orthosurgical treatment not only from a professional point of view, but also from a most important prospect, the patient's.

### Case report 2

**Figure 5:**
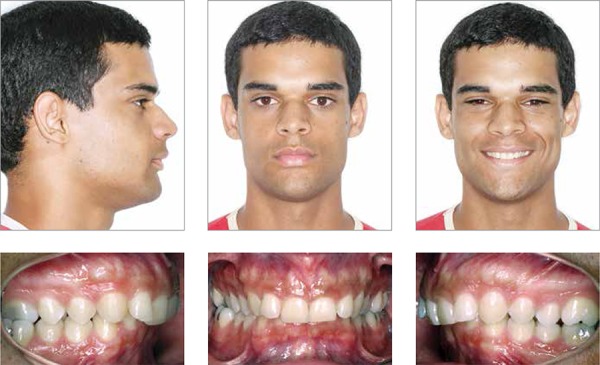
Initial facial and intraoral photographs.

Non-Caucasian male, 26-year-old patient sought treatment complaining about his dental esthetics. As shown by his initial records (Fig 5), he presented Class II malocclusion, mild mandibular retrognathia and +6 mm overjet. He was considered a borderline case for which both treatment options were available: Orthognathic surgery and orthodontic camouflage. The patient was unsure about which treatment he should choose, but ended up opting for orthosurgical treatment with the expectation that it would yield better results.

His oral health-related quality of life was assessed. He scored 8 points in the B-OQLQ (from 0 to 88 points) and 11 points in the OHIP-14 (from 0 to 56 points), which suggested a minor negative impact on his oral health-related quality of life possibly due to presenting a slightly severe dentofacial deformity. A conventional orthosurgical treatment protocol was planned.

**Figure 6:**
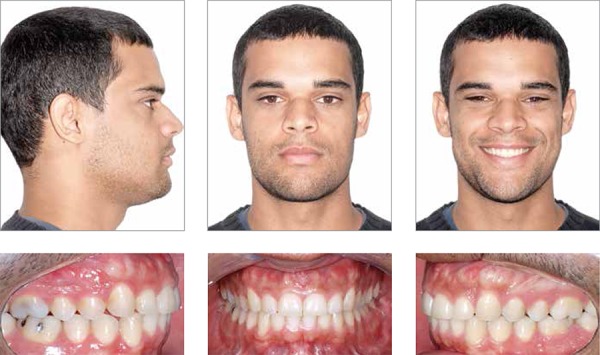
Final facial and intraoral photographs.

An orthodontic appliance was placed and after a preparation that lasted for 2 years and 5 months, mandibular advancement was carried out. The appliance was removed after a 6-month postsurgical orthodontic finishing phase (Fig 6). The patient reported no significant facial alterations and complained about postsurgical discomfort more often than the other patients did. After treatment, he scored 24 points in the OQLQ and 14 points in the OHIP-14, which meant his oral health-related quality of life became slightly worse.

In this case, the surgeon and the orthodontist should have noticed that the patient did not have a full perception of having a mild facial deformity and, for this reason, he did not feel negative impact on his quality of life. Thus, the most appropriate treatment option would be an orthodontic camouflage with the aid of skeletal anchorage. Such protocol could have prevented the orthosurgical treatment from worsening the patient's quality of life.

### Case report 3

**Figure 7:**
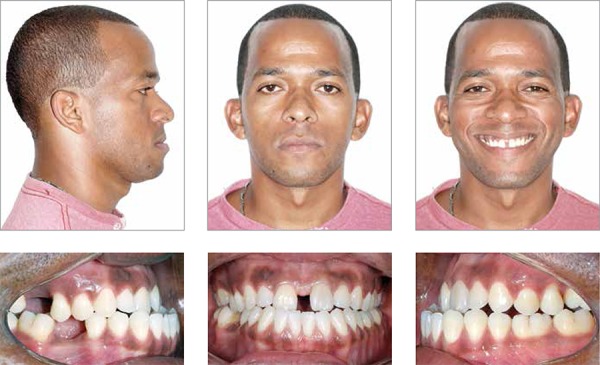
Initial facial and intraoral photographs.

Black male, 32-year-old patient sought treatment complaining about his dentofacial esthetics. He was eager to begin orthosurgical treatment. As shown in his initial records (Fig 7), he presented Class III malocclusion, with severe skeletal discrepancy, maxillary deficiency, mandibular excess and history of extraction of many teeth.

His oral health-related quality of life was assessed. He scored 59 points in the B-OQLQ and 26 points in the OHIP-14, which suggested a considerably negative impact on his oral health-related quality of life, corresponding to the severity of his dentofacial deformity. Due to the great need for orthodontic movement in his case, the conventional orthosurgical treatment protocol was chosen.

**Figure 8:**
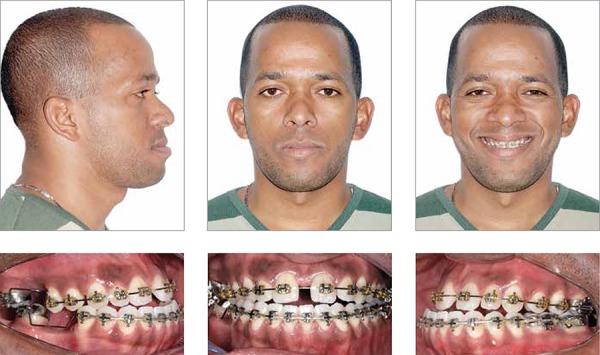
Patient's facial and intraoral photographs in the orthodontic preparation phase.

His B-OQLQ scores were assessed at different periods of orthodontic preparation, a phase the patient is still going through. After one month of orthodontic preparation, an initial reduction in the negative impact on patient's quality of life was observed, with a score of 43 points. Six months after preparation, the patient scored 51 points, suggesting that his condition became slightly worse. After one year of orthodontic preparation, he scored 63 points, an even more negative impact than his initial condition. The patient has been going through orthodontic preparation for two years now (Fig 8) and scores 77 points in OQLQ, thus demonstrating that the longer this treatment phase lasts, the more the patient's quality of life becomes worse due to postponing the benefits produced by esthetic and functional alterations.

These data suggest that there should be a preference for orthosurgical treatment protocols with a substantially reduced preparation phase, such as the anticipated benefit protocol, for patients to feel less negative impact on their quality of life.

## FINAL CONSIDERATIONS

The current objective of orthodontic treatment associated with orthognathic surgery consists not only in treating the esthetic functional components of dentofacial deformities, but also in considering patients' psychological factor. Both are parallelly influenced; but patients' motivations, perceptions and expectations play a significant role in obtaining successful surgical and psychological results.^27^ All these factors influence patients' quality of life. The latter must be assessed by means of previously validated questionnaires, such as the B-OQLQ, before treatment onset, so as to determine the condition's level of influence on patient's quality of life and how such condition can be improved in order to ensure patients' satisfaction, reestablishing his health as a whole and, thus, justifying the biological/financial costs the patient had in both public and private instances.
